# Blood flow restriction pressure recommendations: a tale of two cuffs

**DOI:** 10.3389/fphys.2013.00249

**Published:** 2013-09-10

**Authors:** Jeremy P. Loenneke, Christopher A. Fahs, Lindy M. Rossow, Robert S. Thiebaud, Kevin T. Mattocks, Takashi Abe, Michael G. Bemben

**Affiliations:** ^1^Neuromuscular Research Laboratory, Department of Health and Exercise Science, The University of OklahomaNorman, OK, USA; ^2^Department of Exercise and Sports Science, Fitchburg State UniversityFitchburg, MA, USA; ^3^Department of Health and Physical Activity, University of PittsburghPittsburgh, PA, USA; ^4^Department of Kinesiology, School of Public Health, Indiana UniversityBloomington, IN, USA

**Keywords:** KAATSU, hypertrophy, strength, vascular occlusion training

## Introduction

Blood flow restriction (BFR) alone or in combination with exercise has been shown to result in favorable effects on skeletal muscle function and morphology (Loenneke et al., 2012a). BFR is a stimulus commonly applied with specialized pressure cuffs placed at the top of a limb which are inflated to a set pressure throughout exercise. The pressure applied should be high enough to occlude venous return from the muscle but low enough to maintain arterial inflow into the muscle. Throughout the literature several different methods are applied with respect to setting the BFR pressure, however, many of these appear methodologically flawed. The purpose of the current manuscript is to discuss the importance of setting BFR cuff pressure based on appropriate factors. This manuscript will focus on applying pressures to the lower limbs because the majority of the data has been collected on the lower body.

## Arbitrary pressures

Throughout the literature it is common to have the same BFR pressure applied to each participant, independent of individual differences (Fahs et al., 2012). However, the literature suggests that the pressure applied should largely be dependent upon the width of the cuff applying the stimulus as well as the size of the limb to which the stimulus is applied (Shaw and Murray, 1982; Crenshaw et al., 1988; McEwen et al., 2002; Younger et al., 2004; Loenneke et al., 2012b). When investigations ignore cuff size and/or inter-individual differences in limb size and apply pressures used previously in the literature, it may not only decrease the effectiveness of the intervention, but it may also become a safety concern.

To illustrate, three studies used an arbitrary pressure of 200 mmHg for each individual independent of any other physiologic factor. The first 2 studies applied 200 mmHg using a narrow 5 cm cuff (Fujita et al., 2007; Fry et al., 2010) whereas the most recent study used a wider 11 cm cuff (Gundermann et al., 2012). This is problematic in that the same absolute pressure applied with a wide cuff has been shown to result in differences in arterial occlusion pressure at rest (Loenneke et al., 2012b) and pronounced changes in cardiovascular function when compared to the same pressure applied with a narrow cuff during resistance exercise (Rossow et al., 2012). A dataset from our lab where we have quantified supine arterial occlusion with both 5 and 13.5 cm cuffs can help illustrate the problem with the application of arbitrary pressures. In our narrow (5 cm) cuff dataset of 83 participants, 19 of them would be at or above their respective arterial occlusion pressure at an arbitrary BFR pressure of 200 mmHg. Therefore, it is possible that some of the participants in the previously discussed investigations (Fujita et al., 2007; Fry et al., 2010; Gundermann et al., 2012) were under complete arterial occlusion at rest. Pressures should be relative to the individual just as the loads lifted in those studies were relative to the individual.

## Brachial systolic blood pressure

Several investigations have tried to apply relative pressures based on brachial systolic blood pressure (bSBP; Cook et al., 2007, 2010; Manini et al., 2011; Rossow et al., 2012). For example, in the BFR literature it is common to apply BFR pressure for the lower body based on a percentage of the individuals bSBP (e.g., 130%). Although it may appear to provide a relative method, there is little evidence that bSBP provides a good estimate of BFR to the lower limbs. This lack of relationship between bSBP and lower body arterial occlusion is not surprising given the large differences in limb sizes between the upper and lower body. A recent investigation found that bSBP did not significantly account for any variance in any of the prediction models used to predict lower limb arterial occlusion pressure (Loenneke et al., 2012b). The biggest predictor of arterial occlusion pressure was thigh circumference which is supported by previous surgical literature (Shaw and Murray, 1982; Crenshaw et al., 1988).

Using our lab's aforementioned datasets, applying 130% of bSBP would result in arterial occlusion in 49 out of 116 participants if the investigation were to use a wide 13.5 cm cuff; whereas, only 1 participant out of 83 would be under arterial occlusion if the stimulus was applied using a narrow 5 cm cuff. This highlights the importance of basing pressures on cuff width.

## Possible differences between cuff materials

Many of the narrow cuffs used are made of elastic material whereas the wider cuffs are made of nylon. It is possible that this difference in material may result in differences in in the ability to restrict blood flow and some of this difference may be due to differences in initial pressure. The initial pressure represents the pressure applied to the limb by the elastic cuffs prior to actual inflation (Karabulut et al., 2011). Although not always reported in the literature, it is important to set an appropriate initial pressure prior to inflating the elastic cuffs to the target pressure with the Kaatsu Master/Mini apparatus (Sato Sports Plaza, Tokyo, Japan). The ability to check the initial pressure appears exclusive to the Kaatsu Master/Mini devices. It should also be mentioned that the initial pressures are different between the Kaatsu Master and Kaatsu Mini devices, with the Kaatsu Mini's initial pressure being approximately half that of the Kaatsu Master (unpublished observations).

Recent data suggests that when narrow elastic cuffs (5 cm) are applied at an initial pressure of 50 mmHg and inflated to a target pressure that they restrict blood flow similarly at rest (Loenneke et al., 2013b) and during exercise (Loenneke et al., in press) to narrow nylon cuffs (5 cm) applied to the same target pressure. Taken together it appears beneficial to have the initial pressure standardized to 50 mmHg with the Kaatsu Master and 25 mmHg with the Kaatsu mini as to allow for better comparisons across studies using cuffs of different material.

## Pressure recommendations

In addition to accounting for cuff width, the size of the limb must also be accounted for. To account for inter-individual differences, some investigations have based pressure on a percentage of arterial occlusion pressure (Laurentino et al., 2012). Since the goal of BFR is venous pooling without arterial occlusion, the investigators would then take a percentage of this arterial occlusion pressure as the BFR pressure to use for that individual. While this may be an effective means to set a relative pressure with wider cuffs (13.5 cm cuff), the usefulness of this technique with a smaller cuff is questionable as arterial blood flow may not be able to be occluded with a smaller cuff. Thus, we suggest basing the pressures on the individuals thigh circumference. This method is likely imperfect but does appear to provide a relative BFR stimulus (Loenneke et al., 2013a).

## Conclusions

It is our hope that this manuscript provides further rationale for the importance of basing the BFR pressure not only on the size of the cuff but also making that pressure relative to each individual's limb circumference. These changes need to be made in order to promote not only a more optimal but also a safer stimulus. Without these changes, comparing results between studies becomes almost impossible. We wish to suggest that all future work conducted using BFR make an attempt to make the pressure relative to the width of the cuff as well as to each individual. Further, future studies using BFR should refrain from using arbitrary pressures for everyone or pressures based on bSBP. These recommendations are only applicable to the lower body and may not necessarily reflect what occurs in the upper body.
